# Describing Zoom exhaustion and fatigue in nursing students at a university in South Africa

**DOI:** 10.4102/hsag.v29i0.2675

**Published:** 2024-10-28

**Authors:** Waheedha Emmamally, Dorien Wentzel, Petra Brysiewicz

**Affiliations:** 1 Discipline of Nursing, School of Nursing & Public Health, University of KwaZulu-Natal, Durban, South Africa

**Keywords:** Bachelor of Nursing, Zoom Exhaustion and Fatigue Scale, online teaching, online learning, cross-sectional study, nursing students

## Abstract

**Background:**

Online platforms have gained considerable support from students and lecturers post COVID-19, however they are associated with student exhaustion and fatigue.

**Aim:**

To determine Zoom exhaustion and fatigue in nursing students at a selected university in South Africa.

**Methods:**

A quantitative cross - sectional study was conducted in KwaZulu-Natal to collect data from 146 nursing students registered at the selected university for the 4-year Bachelor of Nursing programme. The Zoom Exhaustion and Fatigue Scale collected data on responses to domains of general fatigue, visual fatigue, social fatigue, motivational fatigue, and emotional fatigue. The data were analysed using the International Business Machines, Statistical Package for Social Sciences, version 26.0. Descriptive statistics were calculated for demographics, a total score was calculated and mean scores and 95% confidence intervals for the different domains were calculated. Mann Whitney U and Kruskal Wallis Independent Tests were calculated to determine associations between demographics and Zoom usage.

**Results:**

The overall Zoom exhaustion and fatigue score was 46.71 (s.d. = 10.50). The motivational fatigue construct had the highest mean score of 3.29 (s.d. = 0.83), followed by general (3.18 s.d. = 0.81), social (3.11, s.d. = 0.88), emotional (3.06, s.d. = 0.99) and visual fatigue (2.92, s.d. = 0.94).

**Conclusion:**

The study concluded that while the overall mean score indicated moderate levels of Zoom exhaustion and fatigue among respondents, majority of the respondents scored high levels (> 48) of Zoom fatigue. Students reported higher levels of motivational fatigue compared to the other 4 constructs.

## Introduction

In January 2020, the World Health Organization (WHO) officially announced the discovery of a novel coronavirus SARS-Cov2, responsible for the infectious respiratory disease called coronavirus diseases 2019 (COVID-19), and declared a pandemic (WHO 2020). The outbreak brought about global disruption to countries’ health, social, economic and educational systems. The need for social distancing because of the pandemic resulted in monumental changes in teaching and learning interactions at educational institutions globally (Barrero, Nicholas Bloom & Davis 2021). In higher education, the traditional classroom teaching and learning pedagogy was transformed overnight to being exclusively remote or online learning with both lecturers and students communicating via various video conferencing platforms (Dhawan 2020; Tempski et al. 2020). Students and lecturers had to quickly gain additional technological expertise and lecturers became innovative in developing online strategies to augment learning opportunities for their students (Guzacheva 2020). South Africa, like most other countries, also remodelled its educational system to embrace various online learning platforms to continue with teaching and learning during the pandemic. However, challenges with using these platforms were encountered. The challenges included connection issues to audio and video, limited access to internet, unreliable internet connectivity, bandwidth and limited or no knowledge of using an online platform (Archibald et al. 2019; Gumede & Badriparsad 2022). These challenges became extremely significant to the quality of teaching and learning on the African continent where technological advancements and technological skills and knowledge may lag behind the rest of the world (Chibuwe & Munoriyarwa 2023).

Nursing students exposed to the online learning platforms faced similar difficulties (Buthelezi & Van Wyk 2020). Agu et al. (2021) highlighted that nursing student socialisation was severely affected by the pandemic, as students would previously meet face to face as groups with their peers and lecturers to discuss class content and gain assistance with new developments and or content in teaching and learning. With the unexpected pandemic, students found themselves isolated and with minimal socialisation with peers and lecturers (Leal Filho et al. 2021). As lectures were now delivered online, this added to nursing student stressors as they were unsure of the type of assessments they would be required to complete. Clinical sessions were suspended, and many final year nursing students were worried if their nursing programme would be extended (Agu et al. 2021).

The rapid transition from physical to digital interactions also raised concerns about the psychological effects of Zoom fatigue, which refers to the feeling of exhaustion or burnout associated with using or participating in video conferencing or virtual meetings through the Zoom platform (Bennett et al. 2021; Fauville et al. 2021a). Zoom fatigue may be caused by the complexity of the specific spatial dynamics taking place in video conferences or by the additional cognitive effort needed to interact with others in this context (Fauville et al. 2021b), namely increased screen time, reduced non-verbal cues and the need to constantly remain engaged. The presence of Zoom fatigue was significant to the quality of teaching and learning that was undertaken at tertiary institutions in SA as most adopted Zoom as their preferred platform of teaching and learning during the pandemic. Furthermore, tertiary educational institutions having identified the flexibility of video conferencing student–lecturer interactions may well continue using the platform beyond the pandemic. There was therefore a need for educators to be knowledgeable regarding the utilisation of video conferencing or Zoom platforms and possible disadvantages thereof (Bailenson 2021; Bennett et al. 2021). It also became imperative for universities to recognise the threat of fatigue and exhaustion stemming from online teaching and learning and plan strategies to reduce or alleviate it. The rationale for the study stemmed from these premises.

### Study aim and objectives

The aim of the study was to determine Zoom exhaustion and fatigue in nursing students at a selected university in KwaZulu-Natal (KZN). The objectives were to describe the levels of Zoom exhaustion and fatigue by the domains of general, visual, motivational, emotional and social fatigue and to test the associations between demographics and use of video conferencing and exhaustion.

## Research methods and design

### Setting

The study was conducted within the Discipline of Nursing at a selected university in the eThekwini District in KZN. During the COVID-19 pandemic, teaching and learning at the university was conducted exclusively on–line platforms, with Zoom meetings being the primary medium of teaching and learning.

### Study design

A cross-sectional, online survey was conducted among nursing students at a selected university in KZN.

### Population and sample

A census was used where the entire population of nursing students in the programme, years 1 to 4 (*N* = 321) were invited to participate. Year 1 comprised of 81 students and years 2, 3 and 4 comprised of 80 students each. The criteria for inclusion were being ≥ 18 years; registered for the nursing academic year 2020 and 2021 and willing to participate in the study. Nursing students who did not meet the criteria were excluded from the study.

### Research instrument

An electronic survey questionnaire using Google Forms was used. The questionnaire comprised Sections A (demographic variables) and B of the Zoom Exhaustion and Fatigue Scale (ZEF scale) was created on SurveyMonkey. The demographic variables consisted of the level of study, age, gender, frequency of participating in video conferencing, frequency of using a desk that allows for the student to stand, the device used for video conferencing in a week and the average duration of a video conference call.

The ZEF scale, used with permission from the developers (Fauville et al. 2021a), measures five constructs of Zoom exhaustion and fatigue, namely general fatigue, visual fatigue, social fatigue, motivational fatigue and emotional fatigue. The scale has 15 items, with 13 items being scored on a 5-point Likert scale, ranging from: 1 = Not at all, 2 = Slightly, 3 = Moderately, 4 = Very and 5 = Extremely. Two survey items: ‘I don’t feel like doing anything’ and ‘I often feel too tired to do other things’ are scored from: 1 = Never, 2 = Rarely, 3 = Sometimes, 4 = Often and 5 = Always. Each construct has a score interval between 3 (low fatigue) and 15 (high fatigue). The Zoom exhaustion and fatigue score is the averaged rating across the 15 fatigue items and ranges from 15 (less fatigued) to 75 (more fatigued) with 37.5 being the median (Fauville et al. 2021a). In addition, respondent scores are divided into low and high fatigue levels according to the median. High Zoom fatigue levels are considered when the overall Zoom exhaustion fatigue score is ≥ the median (Ghanem, Elhussiney & Elbadawy 2023).

The ZEF scale has a high reliability score of *α* = 0.95 (Fauville et al. 2021a), and in this study, the reliability of the ZEF scale was determined by measuring the internal consistency using a Cronbach’s alpha coefficient (*ɑ* = 0.96). A pilot study was conducted with five nursing students, who were conveniently sampled and not included in the analysis. The 15 items and the ZEF scale was found to be easy to read and understand. The researchers ensured the face validity of the scale as they are all lecturers in the BN programme.

### Data collection procedure

Data collection occurred between July and September 2021 after receiving gatekeeper permission and ethical approval. The nursing students of each year were recruited during their online lecture after the researchers had liaised with the respective lecturers to address the students. The questionnaires were distributed via an online link on the Moodle platform (official teaching and learning platform of the university), which directed the students to the Google Forms questionnaire. The students were informed that the online questionnaire would close on 30 September 2021.

### Data analysis

The data responses from SurveyMonkey were exported and analysed using the International Business Machines (IBM) Statistical Package for Social Sciences (SPSS) version 26.0. The quantitative data were described using descriptive statistics of frequencies, percentages, means and standard deviations (s.d.). Zoom exhaustion and fatigue levels were calculated. High Zoom fatigue levels are considered when the overall Zoom exhaustion and fatigue score is ≥ the median (Ghanem et al. 2023). Mean score and confidence intervals were calculated for the separate constructs. Kruskal–Wallis and Mann–Whitney U independent samples tests were used to determine associations between the respondents’ demographic variables and Zoom usage and their overall Zoon exhaustion and fatigue scores. A *p*-value of < 0.05 was significant.

### Ethical considerations

This research study obtained ethical clearance from the research committee of the University of KwaZulu-Natal where the study was conducted (HSSREC/00003201/2021). Special consideration was given to the right to privacy, confidentiality and anonymity of the study sample. The researchers explained the aim of the study, how to access the electronic survey link and the ethical considerations of confidentiality, anonymity, voluntary participation and consent. As proof of their consent, respondents had to click, ‘Yes, I am willing to participate in the study’, before they could proceed to the survey. Students had the right to withdraw from the research study at any time by logging off. The students were made aware that once the questionnaire was submitted online, withdrawal from the study was no longer possible because of the anonymity of the responses.

## Results

A total of 316 nursing students were invited to participate in the survey and 148 responses were received resulting in a response rate of 47.4%. Two respondents were in the 4th year and were excluded from the analysis (*n* = 146).

### Demographics

The mean age of the respondents was 19.8 years, with females comprising three quarter of the sample (*n* = 116, 79.5%). Nearly half of the respondents were in the 1st year of study (*n* = 69; 47.3%) (Table 1).

**TABLE 1:** Demographic characteristics of the respondents (*N* = 146).

Demographic variables	Frequency	
	*n*	%
**Level of study**		
First year	69	47.3
Second year	44	30.1
Third year	33	22.6
**Age (years)**		
18–19	77	52.7
20–24	65	44.5
25–29	3	2.1
**Gender**		
Female	116	79.5
Male	30	20.5
**Frequency of participating in video conferencing?**		
Once a week	13	8.9
Once a day	9	6.2
Multiple times a day	124	84.9
**Video conference device (typical week):**		
All taken from a computer	20	13.7
Mostly taken from a computer	87	59.6
Half taken from a computer and the other half taken from a mobile device (tablet, phone)	27	18.5
Mostly taken from a mobile device (tablet, phone)	7	4.8
All taken from a mobile device (tablet, phone)	5	3.4
**Duration typical video conference**		
< 30 min	3	2.1
30–45 min	38	26.0
45 min	104	71.2

Note: Age (Mean = 19.8 years; standard deviation = 1.8).

### Zoom usage

Most of the respondents (*n* = 124; 84.9%) reported that they participated in video conferencing multiple times a day and 104 (71.2%) had conferences lasting 45 min or more. Concerning the devices used for video conferencing in a typical week, 87 respondents (59.6%) used a computer.

### Zoom exhaustion and fatigue scores

**FIGURE 1:**
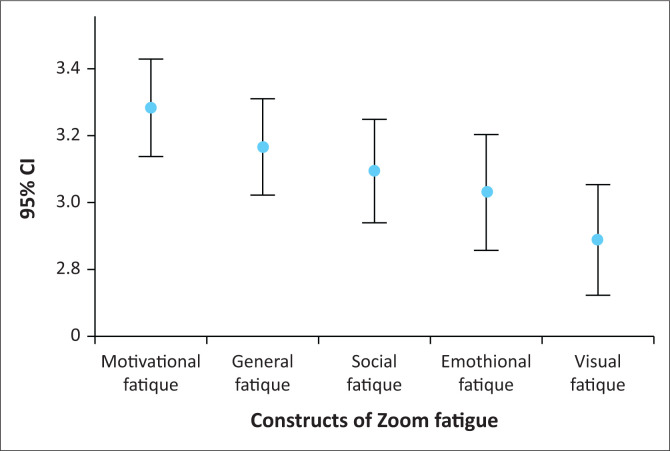
Zoom exhaustion fatigue constructs means and confidence intervals.

The overall Zoom exhaustion and fatigue score for the respondents was 46.71 (s.d. = 10.50) (median = 48.0, mean 3.12, s.d. = 0.70). Just less than half of the respondents, 48.6% (*n* = 71) experienced high levels of Zoom fatigue (> 48) and 51.4% (*n* = 75) experienced low levels of Zoom fatigue (≤ 48) (Figure 1). The motivational fatigue construct had the highest mean score of 3.29 (s.d. = 0.83), followed by general (3.18, s.d. = 0.81), social (3.11, s.d. = 0.88), emotional (3.06, s.d. = 0.99) and visual fatigue (2.92, s.d. = 0.94) (Table 2).

**TABLE 2:** Responses to items and domains (*N* = 146).

Items in the ZEF scale	Mean	s.d.
**Motivational fatigue**	**3.29**	**0.83**
Dread having to do things after video conferencing	3.34	0.94
Feel like doing nothing after video conferencing	3.29	0.99
Feel too tired to do other things after videoconferencing	3.25	0.98
**General fatigue**	**3.18**	**0.81**
Mentally drained after video conferencing	3.27	0.95
Tired after video conferencing	3.17	0.94
Exhausted after video conferencing	3.10	0.92
**Social fatigue**	**3.11**	**0.88**
Need time by yourself after video conferencing	3.22	1.02
Want to be alone after video conferencing	3.13	1.13
Avoid social situations after video conferencing	3.00	1.02
**Emotional fatigue**	**3.06**	**0.99**
Emotionally drained after video conferencing	3.10	1.11
Irritable after video conferencing	3.09	1.12
Moody after video conferencing	2.98	1.12
**Visual fatigue**	**2.92**	**0.94**
Irritated eyes after video conferencing	2.97	1.04
Blurred vision after video conferencing	2.92	1.06
Hurt eyes after video conferencing	2.88	1.06

Note: The bold values represent the constructs of the scale.

ZEF scale, Zoom Exhaustion and Fatigue scale; s.d., standard deviation.

### Associations between the demographic variables and the overall Zoom exhaustion and fatigue levels

No significant differences were found between demographics and Zoom exhaustion and fatigue levels although females reported higher motivational fatigue than males (3.37, s.d. = 0.82 vs. 2.98, s.d. = 0.80, *U = 2.5, p* = 0.011). However, frequency per day had significantly higher scores in the overall score with respondents who participated in video conferencing multiple times a day had higher overall scores (47.9, s.d. = 9.8) than those who participated once a day (35.3. s.d. = 12.1) (*K* = 2.9, *p* = 0.003) or once a week (41.1, s.d. = 10.6), (*K* = 2.2, *p* = 0.022). This finding was also true for all the domains, except for motivational fatigue, across all frequencies of use (*K* = 4.3, *p* = 0.121).

## Discussion

The study aimed to conduct a cross-sectional survey of Zoom exhaustion and fatigue among nursing students’, specifically determining the overall Zoom exhaustion and fatigue scores and looking at the associations between the demographic variables and Zoom exhaustion and fatigue scores of these students.

As shown by the demographic profile, 52.7% of the nursing students were between 18 and 19 years, with majority of the nursing students (79.5%) being females. These results are important to consider as other studies have concluded that younger females are more prone to Zoom fatigue (Döring et al. 2022; Ghanem et al. 2023; Montag et al. 2022). Puba and Amila (2022) suggested that females tended to be more anxious, and this could possibly result in higher stress levels. Fauville et al. (2021b:2) explained that females are more cognisant of facial expressions and emotions of the video conferencing participants; this can lead to them becoming self-conscious thereby exhibiting ‘mirror anxiety’.

Many respondents indicated that they did not use a desk that allowed them to stand up during video conference, and Bennett, Gabriel and Calderwood (2020) suggest that fatigue during video conferencing may be reduced by taking small breaks and by standing up and walking. Nursing students in the study reported that the average video conference lasted 45 min. Oducado, Dequilla and Villaruz (2022) showed how lengthier virtual meetings parallel participants experiencing fatigue. Our respondents also reported that they attended video conferencing multiple times a day, possibly contributing to information explosion (Singh, Steele & Singh 2021). Fauville et al. (2023) posit that longer and more frequent video conference meetings may result in Zoom fatigue. In contrast, Nesher Shoshan and Wehrt (2021) described that irrespective of the number of video conferences attended daily and the length of time of the video conference; participants experienced fatigue. However, Puba and Amila (2022) explain that video conferencing requires more attention by the student, and this, together with lengthy concentration on the learning material, can lead to students feeling exhausted and mentally drained. Lecturers should consider duration and frequency of video conferencing when utilising online learning platforms to decrease Zoom fatigue.

The overall Zoom exhaustion and fatigue score in this study was 46.61, which compared less favourably with a study by Ghanem et al. (2023) where the overall Zoom exhaustion fatigue score was mean = 31.7. However, similar to a study by Oducado et al. (2022), around half of the respondents in this study (48.6%) experienced high levels (> 48) of Zoom fatigue. A study by Ebardo, Padagas and Traper (2021) revealed that students experiencing moderate to high levels of Zoom fatigue are prone to stress and apathy, corroborating the results of our study as students reported moderate levels of feeling drained, irritable and being moody.

In further analysis of the Zoom exhaustion and fatigue scores of the five constructs, motivational fatigue was rated the highest, followed by the social fatigue. Conversely, Oducado et al. (2022) in their study in the Philippines reported the highest mean Zoom exhaustion and fatigue scores in the visual and general fatigue constructs. Utilising a 3-construct scale developed from the ZEF scale, Mariappan and Nordin (2021) concluded that physical, emotional and mental fatigue were equally experienced by Malaysian students during video conferencing. Using the Media Naturalness Theory, Riedl (2022) reinforces that humans need physical contact interactions where they can visualise and hear each other, maintain eye contact and observe facial expressions and body language. The author adds that video conferencing lacks the characteristic of natural communication, which may then progress to Zoom fatigue. This study result may point to the need for lecturers to incorporate features of breakaway rooms and online chats that further facilitates students’ discussion and feedback.

The study found a significant association between participation in video conferencing on multiple occasions per day and Zoom fatigue (*p* = 0.003), and this is in keeping with a study by Elbogen et al. (2020). These findings could be explained by the fact that the cognitive load is significantly increased in video conferencing. The results emphasise the need for lecturers to coordinate online timetables to avoid students attending multiple video conferences in one day.

### Limitations

A limitation that is worth noting is that the survey relied on self-reports, and the results may have been subject to recall biases. In addition, the researchers were also known to the students and were involved in their educational programme at various levels. The study results are limited to a small study sample and one setting, which restricts the generalisation of results.

### Recommendations

Given the continued use of virtual platforms for teaching and learning, it is important for educators to be aware of the potential for exhaustion and fatigue arising in students. Efforts need to be made to avoid students attending multiple video conferences in one day and investment into learning strategies that keep students engaged. Future research into Zoom exhaustion and fatigue should involve multiple settings and analysis be conducted to compare results across different levels of students.

## Conclusion

The aim of the study was to conduct a descriptive survey of Zoom exhaustion and fatigue in nursing students at the selected university in KZN, South Africa. The results of the study mimicked the results of most studies using the ZEF scale, in that the majority of the nursing students had high levels of Zoom fatigue (> 48) and motivational fatigue had the highest Zoom exhaustion and fatigue score among the five constructs. A positive correlation was noted between the overall Zoom exhaustion and fatigue score and the number of video conferences that students attended for a day. It is important that lecturers who continue to use video conferencing as the medium for teaching and learning use evidence-based strategies to negate the effects of Zoom fatigue and keep students actively involved in conferencing sessions.
